# Stakeholder understanding of social prescribing in England: a qualitative study in primary care

**DOI:** 10.1186/s12875-025-02908-9

**Published:** 2025-07-23

**Authors:** I. Fattorini, D. Westlake, A. Turk, K. R. Mahtani, S. Tierney

**Affiliations:** University of Oxford, Oxford, UK

**Keywords:** Link workers, Qualitative research, Semi-strucutred interviews, Social prescribing, Thematic analysis

## Abstract

**Background:**

Social prescribing (SP) seeks to support patients’ wider needs by connecting them to non-medical community resources. Link workers (LWs) facilitate SP’s delivery across the National Health Service (NHS) in England. As a concept, SP may be perceived in different ways by various stakeholders. This study set out to explore how SP is understood among healthcare professionals (HCPs), voluntary and community sector (VCS) representatives, LWs, and patients (Ps) in England.

**Methods:**

A secondary qualitative analysis was conducted using interview data from a realist evaluation on the implementation of LWs in primary care. Interview data from 106 participants (HCPs, VCS representatives, LWs, Ps), across seven sites in England, were analysed using reflexive thematic analysis.

**Results:**

Analysis resulted in 127 codes. These were clustered into the following themes: (1) the need for system optimisation, (2) SP as a tool for personal empowerment, (3) SP’s broad and inclusive nature, (4) community engagement through LWs, and (5) a holistic approach to well-being. These themes highlight SP’s potential as an integrated and empowering ecosystem; requiring effective collaboration and clearer communication among stakeholders to enhance understanding of its purpose, streamline referral processes, and align expectations for greater impact. Understanding of SP could be related to five broad questions around how, who, what, where, and why; the themes produced from the analysis aligned with these questions, each exploring different dimensions of SP. Through this, we developed the 5Ws Framework, which is outlined in the paper.

**Conclusions:**

SP is not a standalone intervention; it is a complex system that requires optimisation and balance across its elements. Its effectiveness as an integrated empowerment ecosystem depends on addressing all facets of the 5Ws—how, who, what, where, and why it operates—engaging the right stakeholders, clearly defining its scope, and implementing it appropriately. Policymakers and commissioners could use the 5Ws Framework to guide decision-making, align health system priorities, and ensure the effective integration of SP within primary care.

**Supplementary Information:**

The online version contains supplementary material available at 10.1186/s12875-025-02908-9.

## Background

Healthcare systems face immense pressure due to surging demand, challenging working conditions, and systemic operational issues [1]. These pressures are compounded by staff retention challenges, workforce shortages, and an ageing population [2, 3]. Burnout, high turnover rates of staff, and inadequate support reduce care quality and continuity [4].

Traditional medical models may fail to address non-medical factors impacting health outcomes. Wider determinants of health - such as loneliness, low income, housing issues, and education - affect behaviours like diet, exercise, and mental health, accounting for 30–55% of health outcomes [5]. These unmet needs further strain healthcare systems, necessitating innovative solutions to improve health delivery.

In England, the National Health Service (NHS) adopted social prescribing (SP) in 2019 [6] to enhance primary care sustainability. SP is gaining global recognition as a promising method to address wider determinants of health by connecting patients with non-medical services or support [7]. It is a central element of NHS England’s Personalised Care initiative, funded through the Additional Roles Reimbursement Scheme (ARRS) [8], which was introduced to improve access in primary care by having a range of new staff contributing to its skills mix.

As a key component of SP, link workers (LWs) are employed to help individuals access community-based services, to support their broader health and well-being [9]. In England, it tends to be General Practitioners (GPs) referring patients to a LW; this individual will then connect patients to relevant support, such as housing advice, creative activities or lifestyle programmes [10]. SP services include (but are not limited to) singing/music, gardening, befriending, cooking, and sports [11].

LWs tend to engage in more than just ‘signposting’ people to local services [12]. Research has shown the complexity of their role, which involves taking time to understand the patient and their well-being goals and having up-to-date knowledge of local resources (e.g. groups, organisations, charities) [13–15].﻿ They have to be sensitive to patients’ readiness to engage; knowing when to gently encourage them to move forwards and when to hold back and, instead, adopt an anchoring role whereby they help people to establish a state of stability, giving them permission to discuss their difficulties and knowing at what point to encourage them to consider potential solutions, when relevant, often in the local community [16].

SP extends healthcare delivery from traditional clinical settings to community-based interventions. This shift is proposed to reduce health system pressures, achieve cost savings, and empower patients to manage their overall well-being [5, 17–19]. However, SP’s potential can be undermined by limited understanding, among healthcare professionals (HCPs) and patients, about its purpose and scope [20, 21]. Research indicates that HCPs, including GPs, may not fully comprehend SP, leading to underutilisation and inappropriate referrals [22]. This lack of understanding affects how GPs describe SP to patients, reducing patient engagement and stakeholder support [23–25]. Increasing awareness and understanding of SP is vital for its success [26, 27]. Previous studies have focused on operational aspects rather than nuanced stakeholder understanding [7, 23, 28]. Addressing this gap, as described in this paper, is critical to overcoming barriers to implementation and buy-in to SP as a way of supporting patients’ wider needs [29].

## Methods

### Research question

The study set out to address the question: *What is the understanding of social prescribing (SP) in primary care among healthcare professionals (HCPs), voluntary-community sector (VCS) representatives, link workers (LWs), and patients (Ps) in England?*

### Design

The study was a secondary analysis of qualitative data collected from a larger realist evaluation on the role of LWs in primary care [25]. Different terms, such as social prescribers, community connectors, and well-being workers, may be used for LWs [30]. This paper employs the term ‘link worker’, as used by NHS England [31]. The protocol for this secondary qualitative analysis was published at DOI 10.6084/m9.figshare.25872541.

### Setting, sample and data collection

Data were collected between November 2021-November 2022 from seven different locations in England. Part of data collection for the larger realist evaluation involved semi-structured interviews with patients who had seen a LW (*n*=61), LWs (*n*=12), HCPs (*n*=61) and members of the VCS (*n*=20). A copy of questions asked in the overall interviews can be found in supplementary file 1. All interviewees provided informed consent to take part and for their interview to be audio-recorded and transcribed verbatim. Interviews lasted between 20–65 minutes and were conducted in-person or remotely by four interviewers - two male and two female. As part of the wider interview, participants were asked ‘What does SP mean to you?’ Interviewees’ responses to this question were used for this secondary qualitative analysis. The research team placed these sections of interview transcripts into a NVivo file ready for analysis.

### Analysis

This secondary qualitative analysis was directed by the first author of the paper; other authors provided feedback and insights as the analysis progressed. Data were managed in NVivo software and interpreted using reflexive thematic analysis [32], involving the following steps:

1. Familiarisation with data: Analysis began by reading extracted data multiple times. During this process, notes were made as annotations in NVivo and reflections in a Word document. This helped with grasping breadth and depth of data.
2. Coding: Coding in NVivo started by highlighting text segments from the relevant excerpts and creating code labels. Codes were developed from the data and organised into a mind-map using the Miro application. Analysis alternated between using the Miro mind-map and NVivo, with the former providing the overarching view of the code labels and the latter allowing for a deeper analysis of specific codes by re-reading entire interview excerpts when needed.
3. Generating themes: Codes were reviewed to identify patterns. This helped to organise data into broader categories and capture more nuanced meanings.
4. Developing and reviewing themes: When defining and analysing themes, the following questions were addressed [32]:
  - What is the theme about?
  - What is the boundary of the theme?
  - What is unique and specific to the theme?
  - What does each theme contribute to the overall analysis?
5. Refining and naming themes: Once themes were developed, each was named. A definition was developed around what each theme encompassed, supported by quotations from the data. Additionally, illustrative codes were used to capture recurring patterns within the dataset, serving as foundational elements in the progression from code labels to refined themes.
6. Writing up: Each theme was presented in relation to the research question. The analysis included sub-themes and central organising concepts for each theme, supported by quotations from interviewees.

### Reflexivity

The first author, who led the analysis, had a medical background before transitioning to executive roles in healthcare business and administration across global systems. Experiencing healthcare as a practitioner, administrator, and patient, shaped her understanding of its limitations and the importance of humanities in health. Aware of the system’s focus on biomedical solutions over holistic care, the first author used reflexivity to examine the data critically. She was supported in this work by researchers experienced in studying SP, and in qualitative research and implementation sciences. This helped broaden and balance the analysis. These other members of the team had been involved in the larger realist evaluation.

## Results

Data excerpts came from 106 participants interviewed for the main realist evaluation (not every interviewee talked about the meaning of SP for them). Table 1 presents these participants' characteristics.

**Table 1:** Participants’ characteristics

Participant type (*N* = 106)	*P*	19
	HCP	62 (GP = 19)
	LW	12
	VCS	13
Age range	20–85 years old	
Gender	Female	79
	Male	27

From the data, 127 codes were created. Table 2 shows the progression from codes to sub-themes, themes and the overarching theme. It includes each theme’s central organising concept. The five themes developed from the analysis are described below.

**Table 2:** Summarising how the analysis moved from codes to the overarching theme

Overarching theme: Integrated empowerment ecosystem					
Themes	System optimisation	You can do it Be the agent of change	Catch-all. Bring it on	Unity through community. Safe nexus	Wholeness. I am more than my body
Central organising concept	System-wide coordination and communication challenges, rather than inherent issues with social prescribing itself, can hinder its understanding and efficiency	Social prescribing can empower individuals to cultivate more autonomy and resilience in managing their own health and well-being	Social prescribing providing universal access to personalised support: anytime, anywhere and for anyone	Link workers bridge the gap between the healthcare system and community, representing a unique go-to point of care	Emphasizes the view that social prescribing treates individuals as complete beings, acknowledging their physical, spiritual, and emotional dimensions, adding depth to the practice of medicine
Subthemes	*Better messaging* Suboptimal communication and the lack of knowledge undermines the uptake of social prescribing	*An additional resource for GPs* Social prescribing is viewed as a platform for coaching and education, fostering individuals’ (re)discovery of inner strength	*Embracing diversity and inclusivity* Capturing wide array of needs across diverse populations	*Community engagement* The community as a whole transforms into a caregiving entity, while providing a psychologically safe space	*Person-centred care* Social prescribing as a tool to recognize and address unique needs of each person
	*Collaboration. Bridging the gap* Improving coordinating between stakeholders and integrating social prescribing into organisational workflows	*Autonomy and resilience* People are learning to be more independent from the healthcare system	*Multifaceted resources* Offering variety of activities from various domains beyond healthcare	*Role of link workers* Link workers humanize this transformational process	*Holistic approach* Social prescribing demonstrates an intention to care on all levels of existence
Illustrative codes	• Deeper knowledge about community resources • Coordination • Core service delivery • Organised system which recognizes value of community • Coordination • Volunteers working with social prescribing network • Matchmaking process • Lack of time • Lack of capacity • Stopping people falling through the cracks • Task-shifting from GPs to link workers • Feeling too clinical and directive • Never heard of it • Embedded service	• Support continuum • Coaching and education • Social re-integration • Social linking • ‘De-pathologizing’ service • Self-help • Setting up expectations • Resilience • Relief • Relating to people with same issues • Referring to sports teams and groups • Out of the box thinking • Addressing independence • Empowering GPs	• ‘It is for young people as well’ • Tool for all generations • Cross-sectoral model of care • ‘Opening the world’: there is much more out there • Practice with new wings • Access to arts, crafts, culture and museums • Uptake by affluent population as well • Meets unmet needs • Tackling health inequalities • Addressing specific needs and challenges • Prevention • Panacea for all the gaps in primary care	• Replacing home • Replacing family • Non-judgemental space • Signposting to community • Building relationship and trust • Working together to think about the right service • Helping to change life • Addressing loneliness • Having someone to talk to • Being listened to • Filling the gap • Connecting to social workers • ‘Asset based community development’ • We are in this together • Helps navigating life • Community engagement	• Healthcare is dehumanised • Empathy • Doing what feels right • Feeling positive • Emotionally demanding • Additional tool to support and help people • Mental health • De-medicalisation • Holistic model of care • Personalised approach • Non-medical alternatives • Making patients feel better • Working at a pace of patient • Well-being • Lifestyle • Sense of purpose
Addressing various angles of inquiry about the meaning of SP					

### Theme 1 – System optimisation

This theme delved into the complexities surrounding SP as part of healthcare. Data indicated that while participants perceived SP as positively impacting healthcare, its effectiveness could be impeded by inefficiencies in system-wide processes. These processes predominantly stemmed from how messages about SP were communicated and how diverse stakeholders were coordinated.

#### Sub-theme 1.1 – better messaging

Participants across the dataset expressed dissatisfaction with the term SP. Most felt it lacked clarity and could be misleading. The word ‘social’ was said to imply a connection with social services, whilst the term ‘prescribing’ carried connotations of medical or pharmacological interventions, suggesting tangible solutions or treatments would be provided. Messaging about SP was not solely hindered by wording but also by the lack of clarity in explaining what it entailed:

*Site 1, VCS02: I do think some greater visibility around social prescribing defining a little better who they are and what their criteria is, who they see, what are their process and how they actually go about making referrals so that everyone is clear and there is consistency across the service, I think would be a useful thing to have.*

Data confirmed that information about SP was distributed through multiple channels, like leaflets, media, and the Internet. However, participants stated that the general public (and some HCPs) lacked clarity on what SP involved. This could mean that rather than streamlining their workflow, clarifying what SP entailed could take up HCPs’ time. Data suggested that improved messaging could alleviate this burden and ensure that patients had a realistic understanding of SP:

*Site 6, HCP07: The word ‘social prescribing’, you have to explain what that actually means… I just basically tell them we've got people that they're not doctors or nurses, but they’re people there to help and is it something that you're basically interested in.*

#### Sub-theme 1.2 – collaboration, bridging the gap

GPs recognised that a key cause of inefficiency in providing care was lack of capacity to address the various problems with which patients presented:

*Site 3, HCP08: …there’s this mountain of need that we can’t fully address…because we don’t have enough time, and also…we probably don’t have the right skills and knowledge…*

Data suggested that better integration of LWs into the healthcare system (e.g. by providing them with space in primary care to see patients, or giving them a comprehensive induction, or encouraging HCPs to shadow them) could improve performance and bridge existing gaps. Initially unaware of SP and the LW role, some GPs developed understanding and trust in these employees. LWs acknowledged the time constraints GPs faced in addressing patients’ needs and their own ability to assist patients by connecting them with relevant community resources:

*Site 2, LW01: We’ve got more time for them to sit and listen. It’s not the GP’s role really to sit and listen to people’s life stories but it is ours so that obviously is a good thing.*

### Theme 2 – You can do it. Be the agent of change

This theme is related to the proactive role of individual stakeholders, particularly HCPs and patients, in driving transformative change and advocating for SP. It highlights the power of individual agency and leadership in effecting positive shifts and transformative practices and policies within the healthcare system.

#### Sub-theme 2.1 – an additional tool for GPs

Using resources allocated through SP could be perceived as signifying a continuum of support extending beyond the boundaries of the medical system, providing GPs with another means of assisting patients:

*Site 2, VCS03: … we’ve seen many examples where social prescribing would be appropriate. For example, if somebody is living long-term with Bipolar Disorder, or Schizophrenia – they are receiving other types of treatment – they may well also be on long-term medication, but they’re looking for additional support and services.*

#### Sub-theme 2.2 – autonomy and resilience

SP was described by interviewees as enabling patients to become less dependent on a healthcare system and to gain independence in managing daily tasks. Some HCPs regarded SP as a form of coaching, prompting patients to learn about and feel they had agency to engage in self-care:

*Site 1, HCP01: … I’ve seen patients where I’ve medicalised them…I’m not a natural medicaliser and it’s unsatisfactory for both sides because then it doesn’t work … because of SP (patients) have managed to get a job and they’re working in cafes and…they’re contributing to society so now they’re central to the community rather than been a periphery and potentially being a burden. So that’s really empowering and that’s really helped with my health and wellbeing as well to see them benefit this way…*

One clinician (Site 6, HCP14) relayed a story about a woman reliant on a motorised scooter who attended tai chi sessions as part of SP. Over a few months, she transitioned from pain relief and her scooter to using walking sticks, indicating a significant improvement in her health. Patients talked about how SP enhanced their independence, leading to improved self-esteem and confidence in their abilities:

*Site 5, P06: … in all the time I've been in the UK, I’ve never travelled or taken like public transport, and I had to go to Wales for my visa thing. She (LW) got all the maps and the routes and everything for me to help me set things like that. That made such a difference to me, gave me the courage... It’s also not only that, it's just the encouragement and the support to make you feel like you can do more than what you think you can do.*

### Theme 3 – Catch-all. Bring it on

Data suggested that interviewees regarded SP as inclusive, catering to individual preferences and requirements. It could encompass a variety of activities or interventions tailored for different demographics, illustrating how multifaceted community resources can complement conventional healthcare services. This theme underscored the versatility of SP practices, meeting the needs of the broad population seen by LWs, regardless of age or social status. However, the idea of SP being a ‘catch-all’ also carried negative connotations, implying that any issue could be indiscriminately passed on to LWs, who were then expected to handle it.

#### Sub-theme 3.1 – embracing diversity and inclusivity

SP was associated with addressing issues related to social deprivation, ageing, financial struggles and other social determinants of health. In the following excerpt, a GP registrar suggested that SP could serve as a comprehensive solution or a one-stop shop for patients with various challenges, emphasising a broad perspective of what SP could offer:

*Site 5, HCP05: … it’s the everything else that’s going on a little bit more widely… if someone is lonely and needs support, they’re (LWs) a good resource for that… or other problems, gambling, homelessness, lots of different kind of, they’ve got a wealth of other stuff at their fingertips that might not necessarily be as easily accessed or known about by other health professionals…*

One LW talked about how, despite her initial perception of affluence in the area she served, underlying deprivation highlighted the need for inclusive support mechanisms such as SP and ensuring that it reached those who otherwise might be overlooked:

*Site 3, LW01: I wasn’t expecting to have as many housing cases … it was an eye opener because I’m working in the west of the borough that ostensibly is wealthier and it obviously is, however, there’s still a lot of deprivation and people struggling in all sorts of ways and there’s quite a lot of social housing…that was quite a surprise…*

It was noted that young people may not be seen as candidates for SP, but they sometimes needed it the most:

*Site 7, HCP01: I thought it (SP) was all to do with elderly and those who couldn’t manage very well. I never thought of young people or children of a certain age and then as the year went on and we had meetings with (LW), she used to come and say, well actually we’re doing x, y, and Z. So, my referral number has gone up greatly.*

#### Sub-theme 3.2 – multifaceted resources

Initially, several HCPs believed SP was a more directed and prescriptive process, focusing on a simple and narrow set of activities. However, over time, there was a shift in understanding towards SP providing access to multifaceted resources:

*Site 5, HCP01: I think it started off being like this idea of connecting people back into the community, but I think we’ve soon learned that there's a lot more to it now … It's so vast and changeable and you're dealing with people, so every person you deal with has different needs.*

As their knowledge of SP expanded, HCPs became increasingly aware of the richness of community resources. This led to a deeper understanding of how resources previously deemed disconnected from healthcare, such as arts and creativity, could address diverse needs and were accessible to patients through SP. Data highlighted a transition from narrow pharmacological solutions stemming from medical settings towards a broader approach to addressing health and well-being:

*Site 7, HCP10: Social prescribing means prescribing things that people need socially, where they don’t necessarily require medical intervention….*

### Theme 4 – Unity through community. Safe nexus

This theme centres around the pivotal role of LWs as intermediaries between healthcare and the community. Data indicated that LWs not only connected people to community resources but also humanised this process, offering nonjudgmental support.

#### Sub-theme 4.1 – community engagement

The community (e.g. local groups, organisations, charities) was viewed as a valuable resource that LWs accessed, with its diverse array of services seen as an extension of care. Engaging the community was said to lighten GPs’ workload and helped to address wider determinants of health. In particular, loneliness emerged frequently in the dataset. It was described as happening after losing a partner or network of friends or feeling isolated for other reasons. SP reintegrated people into society by assisting them in making connections with others, and providing them with support and encouragement to do so:

*Site 5, LW01: … in a nutshell it’s (SP) about signposting people to activities, services and groups, but to also provide emotional support and to help them feel better …*

#### Sub-theme 4.2 – role of LWs

Patients’ engagement with community support was said to rely on the input of dedicated LWs who had time to listen and the expertise to guide people to suitable resources. A description of the role of LWs, using the ‘biopsychosocial’ framework, was suggested by an interviewee who managed SP services:

*Site 2, VCS01: We tend to have to clarify it in terms of what it [SP] isn't, so we’re not GPs, we’re not clinically trained, we’re not counsellors. Sometimes we talk about … the biopsychosocial model, so sometimes I have explained it to people like, bio, that’s what the GP would be doing, psycho, that would be sort of looking at counselling options and therapeutic things, and then the social is more what we do.*

As mentioned above, the importance of saving GPs time and shifting away from medication-centric approaches was frequently highlighted across the dataset. For this to transpire, LWs required extensive, up-to-date knowledge of local community provision. Data suggested a growing recognition of LWs’ expertise around available resources. This comment from a GP showed the distinct contribution they felt LWs made to patient care:

*Site 3, HCP09: … the fact that I’m referring them on to (LW) for instance makes it feel like I am kind of helping them with that burden… I’m giving it to somebody actually who’s much better at dealing with it… I don’t know about the right services, and I don’t necessarily know the best way to access them.*

However, as the following quotation from a SP manager implied, SP was not always accepted across primary care:

*Site 1, VCS01: Sometimes there is a bit of snobbery within the NHS and I don’t have a medical background, and sometimes you speak to Clinical Directors or doctors and I’m not even a human being because I didn’t go to medical school…*

LWs acknowledged the challenges of SP and their inability, at times, to handle all of a patient’s needs; this could be exhausting for them:

*Site 3, LW01: …yes a lot more full on I suppose or more emotionally demanding and yes, demanding in every way really that I didn’t expect.*

### Theme 5 – Wholeness. I am more than my body

This theme highlights an understanding of SP as personalised and holistic. LWs have the potential to acknowledge individuals in their entirety, encompassing their physical, spiritual and emotional aspects, thus enhancing the practice of medicine. Patients expressed the desire for human-centred and inclusive solutions beyond addressing purely medical or physical problems.

#### Sub-theme 5.1 – person-centred care

A fundamental consensus across the dataset was regarding the organisation of SP and LWs focus on patients’ needs. HCPs underscored the importance of patients sharing their stories, and of attentive and empathetic care provision, facilitated through LWs. SP provided an opportunity for LWs to help just by being present and listening:

*Site 3, HCP06: …(LW) has been really helpful because a lot of it is social in general practice – there’s lots of things that we can’t fix, but actually having someone to talk to is really helpful.*

#### Sub-theme 5.2 – holistic approach

This sub-theme relates to SP’s ability to treat a person as a whole, including the spiritual and emotional dimensions of care. Data highlighted how vulnerable populations could form meaningful connections with LWs, who provided space for individuals to explore and address multiple aspects of their health:

*Site 4, HCP07: …it’s (SP) very much about working at the pace of the patient and really holistically tapping into those different areas, so it might be physical health, it might be mental health, it may be social isolation…*

#### Overarching theme – integrated empowerment ecosystem

Participants’ understanding of SP showed it was regarded as a vehicle for transformation (e.g. in how we perceive health can be augmented, in how we see the role of community in supporting population health, in how patients view their role in maintaining health). Transformation came from integrating and coordinating personal and professional aspects into a cohesive system to ultimately enhance quality of care. This led to the creation of an overarching theme from the data – SP as an ‘Integrated empowerment ecosystem’; it highlights that SP was not perceived as a single intervention but combining multiple options, various stakeholders (HCPs, VCS, LWs and Ps), and including a range of processes. Thus, SP can be seen as a conditional solution in a complex system, suggesting that while it holds great potential, its success depends on effective collaboration and incorporation of stakeholders and services involved. This overarching theme anchors the five themes outlined above, which collectively shed light on understanding of SP.

1. ‘System optimisation’ focuses on enhancing SP’s efficiency by improving systemic processes through better messaging, coordination, and stakeholder collaboration, to create a seamless and effective ecosystem.
2. ‘You can do it. Be the agent of change’ underscores the empowerment of individuals within the ecosystem.
3. ‘Catch-all. Bring it on’ highlights SP's inclusivity, reflecting its role in creating a comprehensive support network within the ecosystem.
4. ‘Unity through community. Safe nexus’ emphasises the importance of HCPs, VCS, LWs, patients and community collaboration in building a cohesive ecosystem.
5. ‘Wholeness. I am more than my body’ reflects SP's holistic approach, recognising the interconnectedness within the ecosystem of physical, mental, and emotional well-being.

## Discussion

This qualitative secondary data analysis from a larger realist evaluation produced five distinctive themes that highlight a multifaceted understanding of SP. The overarching theme, ‘Integrated empowerment ecosystem’, captures the interconnectedness of all themes. Findings underscore participants’ viewpoints of SP as a service that requires optimised systemic processes to enhance efficiency. They also emphasise the importance of emotional support and humanised care through LWs. This knowledge can enhance the planning and implementation of SP in England and inform global SP initiatives [18, 33].

### Comparison with the existing literature

Previous research has identified variability in understanding SP among HCPs, which can impact SP’s effectiveness [20, 21, 27]. Themes outlined above add to this, providing further, more detailed exploration and pinpointing areas for optimisation. For example, HCPs feel responsible when referring patients to services; unclear SP processes (e.g. if HCPs are not familiar with the LW role or do not really understand how this individual could help patients) can make this difficult [34, 35], as HCPs are unsure about whether this is the right path for a patient or if the LW can be trusted. Improved communication is therefore crucial, echoing Holtrop and colleagues [26], who emphasised the importance of ‘mental models’ in overcoming implementation challenges. Evers et al. [36] highlighted significant gaps in SP awareness among GPs across 33 European countries, suggesting enhanced GP engagement could maximise benefits. Our participants identified issues with the term ‘social prescribing’; they felt it could mislead and confuse, concerns shared in the research by Pescheny et al. [37], which emphasised the need for improved terminology and communication to enhance SP’s adoption.

The theme ‘Be the agent of change’ highlighted how SP empowers individuals through resources, knowledge, and support networks, aligning with Tierney et al. [38]. Empowering patients fosters engagement and builds social capital. This study further demonstrated that SP can help patients develop autonomy and resilience.

Community engagement and the critical role of LWs are central to the theme ‘Unity through community. Safe nexus’. This aligns with Frostick and Bertotti [20] and Tierney et al. [25, 39] who emphasised the importance of LWs in connecting patients to community resources. By fostering a sense of community, and providing a safe environment, LWs enhance SP’s effectiveness.

The holistic approach of SP is emphasised in the theme ‘Wholeness. I am more than my body’, recognising people as complex beings with diverse needs. Previous studies highlighted SP’s role in addressing physical, mental, emotional, and social well-being [26, 40]. Empathy has been identified as a key skill enabling LWs to do this effectively [20]. This study’s findings corroborate these insights, showing SP’s commitment to person-centred care [25].

A particularly noteworthy finding emerged from the theme ‘Catch-all. Bring it on’, highlighting SP’s inclusive nature in addressing diverse needs. Many participants perceived SP as predominantly for socially deprived or older individuals, potentially limiting referrals for younger and more affluent populations. However, issues like loneliness and mental health, which SP can address, affect people across demographics [41–44]. The concept of ‘candidacy’ is relevant here, describing how eligibility for healthcare is negotiated between individuals and services [45, 46]. In this case, candidacy might be shaped more by HCPs’ assumptions rather than patients’ own perceptions of eligibility. Misconceptions about SP candidacy may limit its reach. Expanding understanding of SP’s benefits among both professionals and the public could enhance accessibility and inclusivity, aligning with research on social determinants of health and their impact across demographics [47, 48]. This includes ensuring that minority ethnic communities, noted in research to be less likely to engage with SP [34, 35], are supported to be aware of and take part in this non-medical approach to supporting health.

### Implications for practice and policy

Findings from this study provide insights for enhancing the implementation of SP in healthcare systems. To maximise SP’s effectiveness, there is a need to improve communication and coordination among GPs, LWs and VCS organisations to ensure a seamless referral process. Raising awareness among HCPs and patients, through clearer messaging and outreach efforts, could help individuals recognise when SP is relevant to them and how they can access it. Without this awareness, patients may not consider themselves candidates for SP or may underestimate its potential benefits, leading to lower uptake. Expanding outreach efforts is crucial to ensure that SP services are accessible to diverse populations, addressing barriers related to eligibility. Additionally, encouraging cross-sector collaboration and exploring alternative funding strategies will support the long-term sustainability of SP initiatives. Addressing these areas can help SP become a more integrated and enduring component of healthcare systems globally.

The themes we developed could be related to five broad questions (how, who, what, where, why), each exploring different angles of the meaning of SP. These questions emphasise how various dimensions of SP are understood (see Table 3), and what providers and commissioners need to consider when implementing and publicising such non-medical support to patients.

**Table 3:** Questions exploring different angles of SP (see also Table 2)

	**Question**	**Answer**	**Interpretation**
1.	How?	‘Optimise the system’	Change needs to happen on multiple systemic levels to make SP more efficient
2.	Who?	‘You’	Every SP stakeholder can become an agent of change
3.	What?	‘Bring it on’	SP uses numerous community resources to match a wide range of unanswered needs
4.	Where?	‘At the nexus’	LWs are key enablers, ensuring connection between healthcare and community providers
5.	Why?	‘I am more than my body’	SP is holistic, treating the whole person and not just medical conditions

**Fig. 1:**
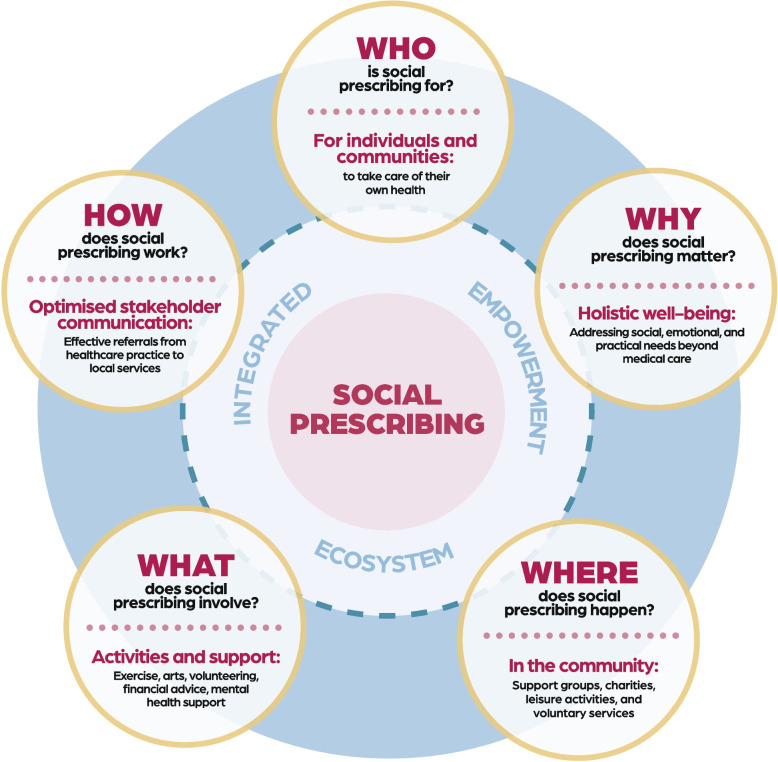
5Ws social prescribing framework: an integrated empowerment ecosystem

Figure 1 helps to visualise the *‘5Ws Social Prescribing Framework: Who, Why, Where, When and How—An Integrated Empowerment System’*. This stakeholder-driven framework relates to findings from Muhl et al.’s Delphi Study [7], which sought to establish a consensus-driven, internationally accepted definition of SP. Our 5Ws Framework provides a bottom-up, real-world perspective that complements the expert consensus approach taken by this earlier Delphi study.

### Implications for future research

Future research should focus on expanding understanding of SP among different stakeholders, including HCPs and patient groups. Investigating new models for integrating SP within primary care settings could improve efficiency and effectiveness. Evaluating SP’s financial sustainability and cost-effectiveness is essential to inform policy decisions and resource allocation. Additionally, research is needed to explore the perspectives of patients who decline referral to SP, identifying barriers such as misconceptions, lack of awareness, or a perceived lack of candidacy. Understanding why some patients do not engage with SP could provide valuable insights into how messaging, referral pathways, and service delivery can be improved. These research priorities will provide insights to guide the development and scaling of SP in healthcare systems.

### Strengths and limitations

The study benefitted from a diverse sample across multiple sites, offering a broad perspective on SP. The use of reflexive thematic analysis allowed for an in-depth exploration of stakeholder perspectives. However, as a secondary data analysis, the study was limited by the scope of the original data collection and may not capture all relevant aspects of SP, including data from patients who did not accept referral to SP. The findings are specific to England, which may limit their applicability to other healthcare systems with different structures and policies. Furthermore, the evolving nature of SP means that recent developments and innovations may not be fully reflected. Despite these limitations, insights gained from this research offer important contributions to the ongoing discourse on SP and its role in healthcare delivery.

## Conclusions

This secondary analysis of qualitative data resulted in an overarching theme – SP as an integrated empowerment ecosystem; this refers to a collaborative and interconnected system where HCPs, LWs, VCS representatives, patients and the wider community work together to support individual well-being. The key topics raised by participants around system optimisation, empowerment, inclusivity, community engagement, and holistic care highlighted the multifaceted nature of SP and its potential to bridge gaps by addressing wider determinants of health. These findings have important implications for practice, emphasising the need for improved coordination, clearer communication, and enhanced support for LWs and patients. For policy, the study underscores the importance of standardised frameworks, sustainable funding models, and cross-sector collaboration to ensure that SP initiatives have long-term success and scalability. The 5Ws framework developed from the data captures the realities and potential of SP as a patient-centred model. This framework emphasises the need for clear definitions and inclusive approaches to enhance SP’s impact.

## Supplementary Information

Supplementary Material 1.

## Data Availability

The datasets generated and analysed during the current study are not publicly available as agreed with the sites involved, but could be available from the corresponding author on reasonable request.

## Abbreviations

GP: General practitioner
HCP: Healthcare professional
LW: Link worker
NHS: National Health Service
P: Patient
SP: Social prescribing
VCS: Voluntary-community sector
