# Supplementary material for: Stakeholder understanding of social prescribing in England: a qualitative study in primary care
Source: BMC Prim Care. 2025 Jul 23;26:230. doi: 10.1186/s12875-025-02908-9 (PMC12285178; doi:10.1186/s12875-025-02908-9)
Supplement: Supplementary file 1 — Supplementary Material 1. [file 12875_2025_2908_MOESM1_ESM.docx]

**Supplementary file 1: Example of questions asked in interviews**

Here are examples of the questions interviewees were asked. However, these differed depending on participants involved and our developing understanding of the topic. We amended the questions asked as data collection advanced. As we progressed the study, we used more of a sense checking approach with interviewees.

| **Patients** | **Link workers** | **Healthcare/VCS staff** |
| --- | --- | --- |
| Can you tell me a bit about how you came to be referred to/meet with [name of link worker]?  *Prompt: What made you decide to see [name of link worker]?*  How did you think the link worker would help you?  *Prompt: How much did you know about this role? Had you heard of link workers beforehand?*  What does the term ‘social prescribing’ mean to you?  Who do you think link workers can help and why?  *Prompt: Any particular groups of patients or problems?*  What sort of things has the link worker referred you to?  *Prompt: How was this decided? What were your thoughts on being linked to these services?*  How satisfied have you been so far in your experiences of the link worker service? | What does the term ‘social prescribing’ mean to you?  Can you tell me a bit about how you came to be working as a link worker?  What were your expectations of the role?  What do you enjoy about being a link worker?  What are the challenges of being a link worker?  How do you work with health colleagues?  How do you work with the voluntary-community sector?  Who do you think social prescribing works for and why?  Who do you think social prescribing doesn’t work for and why?  What do you think needs to be in place/provided for a link worker to be effective? | Can you tell me a bit about the interactions you have with link workers?  What does the term ‘social prescribing’ mean to you?  What do you think about the introduction of link workers into primary care?  How do you think social prescribing and the link worker role is perceived by your colleagues?  Who do you think social prescribing works for and why?  Who do you think social prescribing doesn’t work for and why?  What do you think needs to be in place/provided for a link worker to be effective?  What impact has having a link worker in your practice/area had on your workload? |
